# Comparison of the impact of a system tele-antimicrobial stewardship program on the conversion of intravenous-to-oral antimicrobials in community hospitals

**DOI:** 10.1017/ash.2024.423

**Published:** 2024-10-02

**Authors:** Brenda V. Maldonado Yanez, Kendall E. Ferrara, Richard Lueking, Taylor Morrisette, Erin E. Brewer, Nicole H. Lewis, Rachel Burgoon, Krutika Mediwala Hornback, Aaron C. Hamby

**Affiliations:** 1 Medical University of South Carolina College of Pharmacy, Charleston, SC, USA; 2 Division of Infectious Diseases, Department of Medicine, Medical University of South Carolina, Charleston, SC, USA; 3 Department of Pharmacy Services, Medical University of South Carolina Health, Charleston, SC, USA; 4 Department of Pharmacy Services, Medical University of South Carolina Florence Medical Center, Florence, SC, USA; 5 Department of Medical Education, Quillen College of Medicine, East Tennessee State University, Johnson City, TN, USA

## Abstract

**Objectives:**

Evaluate system-wide antimicrobial stewardship program (ASP) update impact on intravenous (IV)-to-oral (PO) antimicrobial conversion in select community hospitals through pre- and postimplementation trend analysis.

**Methods:**

Retrospective study across seven hospitals: region one (four hospitals, 827 beds) with tele-ASP managed by infectious diseases (ID)-trained pharmacists and region two (three hospitals, 498 beds) without. Data were collected pre- (April 2022–September 2022) and postimplementation (April 2023–September 2023) on nine antimicrobials for the IV to PO days of therapy (DOTs). Antimicrobial administration route and (DOTs)/1,000 patient days were extracted from the electronical medical record (EMR). Primary outcome: reduction in IV DOTs/1,000 patient days. Secondary outcomes: decrease in IV usage via PO:total antimicrobial ratios and cost reduction.

**Results:**

In region one, IV usage decreased from 461 to 209/1,000 patient days (*P* = < .001), while PO usage increased from 289 to 412/1,000 patient days (*P* = < .001). Total antimicrobial use decreased from 750 to 621/1,000 patient days (*P* = < .001). In region two, IV usage decreased from 300 to 243/1,000 patient days (*P* = .005), and PO usage rose from 154 to 198/1,000 patient days (*P* = .031). The PO:total antimicrobial ratios increased in both regions, from .42–.52 to .60–.70 in region one and from .36–.55 to .46–.55 in region two. IV cost savings amounted to $19,359.77 in region one and $4,038.51 in region two.

**Conclusion:**

The ASP intervention improved IV-to-PO conversion rates in both regions, highlighting the contribution of ID-trained pharmacists in enhancing ASP initiatives in region one and suggesting tele-ASP expansion may be beneficial in resource-constrained settings.

## Background

Antimicrobial stewardship programs (ASPs) are integral in addressing the multiple challenges posed by infectious diseases and appropriate antimicrobial usage in a healthcare system.^1^ Among the important initiatives of ASPs, the conversion of IV to PO antimicrobials is a major focal point as it offers the potential to decrease hospitalization length of stays (LOS), reduce healthcare costs, mitigate complications associated with IV antimicrobial use, and improve patient/nursing satisfaction.^2–4^ Inpatient ASPs are guided by the Centers for Disease Control and Prevention (CDC) Core Elements of Hospital Antibiotic Stewardship and leverage the expertise of pharmacists, commonly with formal ID/ASP training, in implementing effective IV-to-PO conversion protocols.^4^ As the healthcare landscape continually transforms, the integration of telemedicine introduces a new dimension to this paradigm. Within this evolving framework, the role of ID-trained pharmacists in telemedicine emerges as a crucial link, offering expertise beyond the confines of physical hospital boundaries and potentially revolutionizing ASPs in resource-limited environments.^5^

Although ASPs have demonstrated considerable success in enhancing antimicrobial use practices, limited data exist regarding the role of telehealth, specifically within the discipline of ASP and its application to IV-to-PO conversion, including optimal models.^6–8^ This emphasizes the need for a focused exploration of telemedicine’s intricate role in enhancing IV-to-PO conversion, an important step toward fortifying the foundations of ASP programs in community hospitals. These hospitals are often faced with the challenge of functioning within resource limitations, which can significantly impact their capacity to establish and maintain effective ASP initiatives.^9^ The potential lack of resources at community hospitals amplifies the importance of tele-ASPs, offering a potentially cost-effective and scalable solution to overcome the limitations imposed by ID-trained specialist shortages and/or restricted budgets.^9^

This study aims to contribute further insights to the tele-ASP literature by evaluating the impact of a system-wide ASP update on the conversion of IV-to-PO antimicrobials in select Medical University of South Carolina (MUSC) community hospitals. Additionally, the study aims to examine the influence of ID-trained pharmacists with dedicated full-time equivalents (FTEs) in tele-ASP on the system-wide ASP update. By evaluating the trends and outcomes, this research seeks to contribute valuable insights into the role of tele-ASP and the added benefit of ID-trained pharmacists in optimizing IV-to-PO conversion practices.

## Methods

This was a retrospective analysis conducted across seven community hospitals within the MUSC Health system, encompassing the period from April 2022 to September 2022 for the preimplementation phase and from April 2023 to September 2023 for the postimplementation phase. In April 2023, an IV-to-PO protocol update was implemented across all of the community hospitals. The comparison time frame from 2022 was selected to account for seasonal variances in antimicrobial usage.

The hospitals were divided into two regions: region one, consisting of four hospitals with a total of 827 beds, and region two, consisting of three hospitals with 498 beds. In region one, oversight of tele-ASP duties was conducted by a pharmacist with Postgraduate Year Two (PGY2) residency training in ID, entailing 12 months of comprehensive training and practical experience across various ID practice domains. This pharmacist was affiliated with the central antimicrobial stewardship team at the flagship institution in Charleston, South Carolina. In contrast, tele-ASP duties in region two were carried out by pharmacists without PGY2 training in ID and with other routine clinical duties. In region one, the ID-trained pharmacist designed and enforced an IV-to-PO list in the electronical medical record (EMR) to alert local pharmacists at each site of potential eligible conversions. Region two also adopted the update in EMR but without an ID-trained pharmacist’s reinforcement.

Nine antimicrobials with high bioavailability were selected based on a preestablished IV-to-PO conversion protocol approved by MUSC Health’s pharmacy and therapeutics committee. The protocol was first launched at the flagship institution and then expanded to the community hospitals. The EMR was updated to alert the local pharmacists if any of the antimicrobials listed in Table 1 could be converted by the criteria listed in Table 2. The local staff pharmacists could automatically convert these antimicrobials to PO if they met the following criteria listed in Table 2. Physicians did not have access to this list.

**Table 1.** AWPs of IV and PO antimicrobials based on dose

Antimicrobial	Dose	IV AWP ($)	PO AWP ($)
Azithromycin	500 mg daily	7.31	3.57
Ciprofloxacin	500 mg twice daily	12.00	5.94
Clindamycin	900 mg three times daily	2.55	1.02
Doxycycline	100 mg twice daily	50.40	8.78
Fluconazole	400 mg daily	46.00	28.72
Levofloxacin	750 mg daily	9.00	36.10
Metronidazole	500 mg three times daily	9.00	2.37
Sulfamethoxazole/ trimethoprim	800/160 mg twice daily	6.08	1.78

**Table 2.** Inclusion and exclusion criteria for MUSC IV-to-PO conversion protocol

Inclusion
1. Functioning gastrointestinal (GI) tract (ie, one of the following) a. Tolerating adequate PO intake (ie, diet ordered or receiving enteral nutrition) b. Receiving other oral medication (s)
2. Resolution/improvement in signs/symptoms: a. Hemodynamically stable b. WBC improving or stable (≤ 15 x 10^3^ /µL) c. Afebrile > 24 hours
**Exclusion**
1. Age < 18 years
2. Patient is being treated for or has the following condition: a. Infection is at a site where an oral agent will NOT achieve adequate concentrations
3. Patient has any of the following conditions: a. Hemodynamic instability requiring high-dose vasopressors b. Active gastrointestinal bleeding c. Continuous enteral nutrition (fluoroquinolones and doxycycline only) d. Continuous nasogastric suctioning e. Grade III or IV mucositis f. Graft versus host disease (GVHD) of the gastrointestinal (GI) tract g. Malabsorption syndrome or documented ileus or gastrointestinal obstruction h. Severe diarrhea (eg, > 5 liquid stools/day) i. Short bowel syndrome j. Uncontrolled vomiting or has received > 2 doses of anti-emetics in the last 24 hours

The inclusion criteria for the protocol was the same as the patient conversion eligibility outlined in the MUSC IV-to-PO conversion protocol (refer to Table 2). Patient demographics were unavailable since the EMR data, sourced from antimicrobial usage reports, does not include this information, and extensive manual chart review was not conducted based on common ASP duties.

The primary outcome was the decrease in days of therapy (DOTs) per 1,000 patient days for the IV formulations of the nine selected antimicrobials. Secondary outcomes included evaluating the decrease in IV usage through PO:total antimicrobial ratios and an assessment of cost reduction in both regions. Data were obtained from the EMR’s antimicrobial usage reports built-in dashboard and recorded as DOT/1,000 patient days for both IV and PO antimicrobials. Subsequently, the data were utilized to compute the PO:total antimicrobial ratios. These ratios were derived by dividing the total PO DOT by the combined total of IV and PO DOT. For visual representation, the ratios were also expressed as percent averages by multiplying the ratio averages by 100%.

For antimicrobial cost analysis, we obtained Wholesale Acquisition Cost (WAC) via Lexidrug® (Table 1).^10^ Since the AWP on Lexidrug® for linezolid did not align within $50 of our WAC pricing, the decision was made by the authors to exclude the antimicrobial from the analysis.^10^ The cost data were calculated by multiplying the total DOT by the AWP price of the medication for both IV and PO formulations. The precost data was then subtracted from the postcost data. The costs are reported as IV cost savings and total cost savings, which is the cost savings of IV and PO combined.

Data analysis involved comparing antibiotic utilization via DOT/1,000 patient days for both IV and PO antimicrobials using IBM SPSS version 28.0. The mean use of each antimicrobial formulation (in DOT/1,000 patient days) was calculated and compared pre- and postimplementation for each region using the Student *t* test. The study did not require approval by an institutional review board, as it was considered a quality improvement initiative for the institution. We submitted it to our local quality improvement board in July 2023, and it was approved as a quality improvement project.

## Results

**Figure 1.**
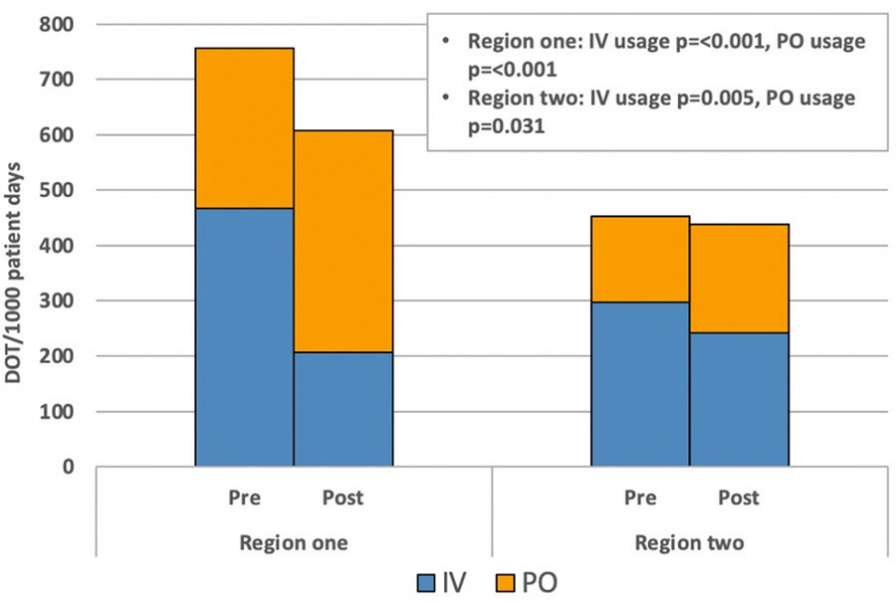
Region one and two median DOT/1,000 patient days for IV and PO usage pre and postimplementation.

**Figure 2.**
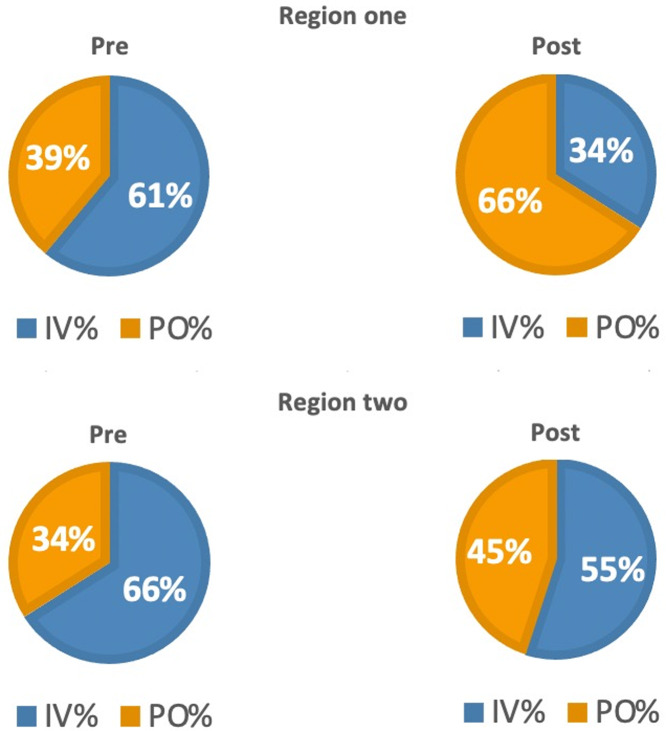
Region one and two average IV and PO usage.

In region one, a statistically significant decrease in the mean DOT for IV usage was observed, decreasing from 461/1,000 patient days in the preimplementation period to 209/1,000 patient days postimplementation (Figure 1). Additionally, there was a concurrent increase in the mean DOT for PO usage, rising from 289/1,000 patient days preimplementation to 412/1,000 patient days postimplementation (Figure 1). This shift was further emphasized by a notable increase in the average percentage of PO use, rising from 39% to 66% during the respective pre and postintervention periods (Figure 2). Following implementation, mean DOT of total antimicrobial use decreased substantially from 750 to 621/1,000 patient days (*P* = < .001).

In region two, the mean DOT for IV usage decreased from 300 to 243/1,000 patient days (Figure 1), while the mean DOT for PO usage rose from 154 to 198/1,000 patient days (Figure 1). This observation translated into the average percentage of PO use in region two rising from 34% to 45% during the pre- and postintervention periods (Figure 2). Following implementation, mean DOT of total antimicrobial use decreased slightly from 454 to 441/1,000 patient days (*P* = .623).

**Figure 3.**
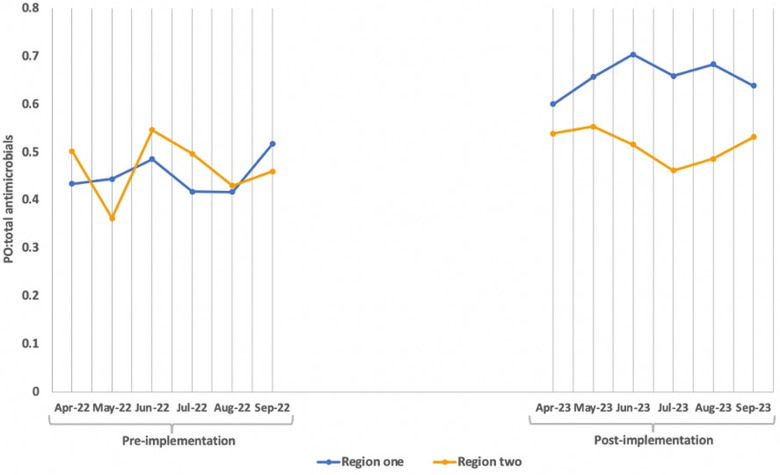
Monthly average PO:total antimicrobial utilization pre and postimplementation.

The examination of average PO:total antimicrobial utilization ratios revealed trends in both regions (Figure 3). In region one, during the preimplementation period, PO:total antimicrobial ratios ranged from approximately .42 to .52, indicative of a moderate oral antimicrobial utilization compared to IV antimicrobials. Following implementation, these ratios increased, ranging from approximately .60 to .70, highlighting a shift toward oral formulations. Conversely, in region two, preimplementation PO:total antimicrobial ratios ranged from approximately .36 to .55, demonstrating a varied usage of oral versus IV antimicrobials. Following implementation, these ratios remained relatively stable, ranging from approximately .46 to .55.

**Figure 4.**
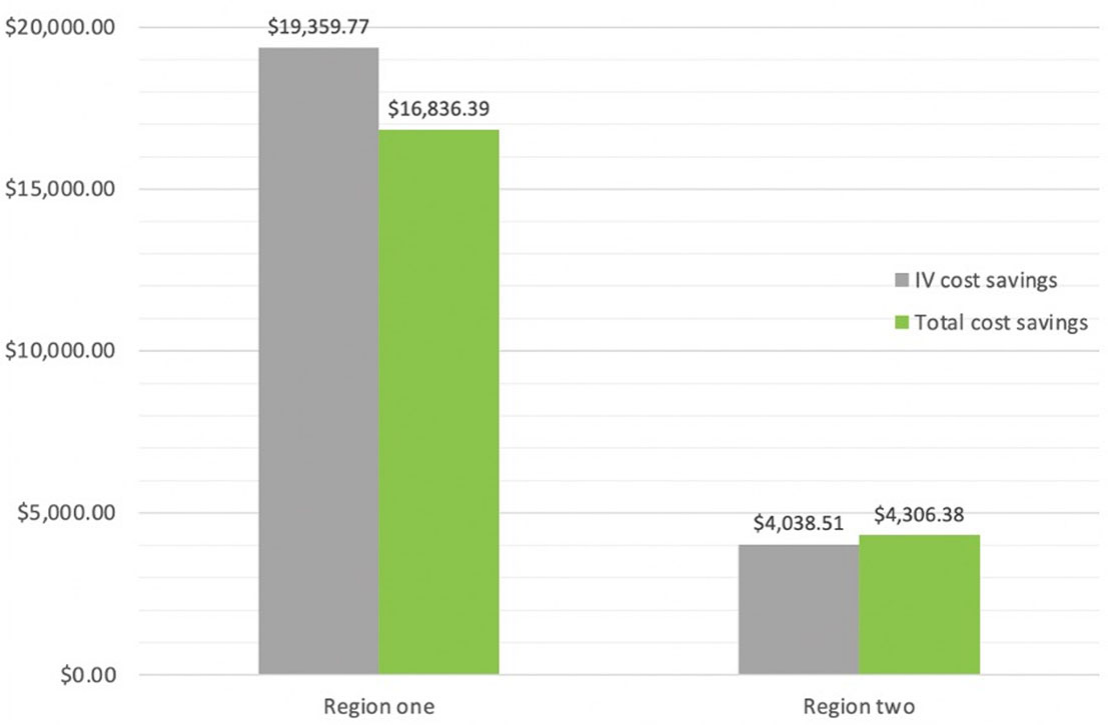
IV and total savings between pre and postintervention periods for region one and two.

The cost analysis showed savings resulting from the IV-to-PO protocol update in both regions. Both regions experienced positive savings, with IV costs reduced and contributing to an overall cost reduction (Figure 4).

## Discussion

Our findings demonstrate the practical implementation of a tele-ASP, but more importantly how to use innovation to leverage an EMR to achieve the ASP mission in a resource limited setting. Since the Joint Commission requires stewardship programs to be in concordance with the CDC Core Elements regardless of hospital size or resources, healthcare systems are tasked to find innovative and creative solutions to meet those requirements, while at the same time seeking to improve patient care and safety.^4,9^ We demonstrate the impact of a specific intervention via a collaborative model as described by Andrzejewski et al.^11,12^ In the first six months of the intervention period, we saw significant improvement in acceptance rates and PO ratios as consistent with other studies at the community level and critical access hospitals.^13–16^

The MUSC tele-ASP began in the fall of 2022 in response to the acquisition of numerous hospitals across the state of South Carolina. The program at the time covered 7 facilities spanning the previously described region 1 and 2 (excluding central academic medical center). Every facility has staff pharmacists with daily responsibilities that include ASP activities. Due to staffing limitations and the timing of mergers, only region 1 had a dedicated ID-trained pharmacist. The ID trained pharmacist coverage is currently Monday through Friday during business hours (8a-5p), but most ASP activities continue over the weekend with staff pharmacists at each facility, including the IV to PO list. The ID pharmacist’s clinical duties include providing oversight and review for all positive sterile site cultures, high risk antimicrobials, and IV to PO de-escalations handled by the staff pharmacists. The ID pharmacist provides prompt education and feedback on the staff pharmacist recommendations. When needed, cross-coverage was provided by ID pharmacists at the main campus. In addition to the 24/7 system-wide ASP steward-on-call, a System ASP Pharmacy Coordinator role was established in March of 2023 to help standardize practices across the enterprise. This role served as an interim point person for Region 2. We have since been able to advocate for two additional FTEs for the enterprise (including Region 2), showcasing our wins with the IV-to-PO initiative. The System ASP physician leadership includes an adult ID physician with director-level duties but minimal daily oversight. Telemedicine ID consultation is available in region 1 and in-person ID consultation is available in region 2; however, these services have limited daily involvement in and commitment to ASP activities. The EMR update was across the system; however, region one had a dedicated ID pharmacist that was consistently looking at the lists daily and communicating with pharmacists to de-escalate IV to PO. In region one, the mean DOT of total antimicrobial use after the intervention was substantially decreased and statistically significant. Region two saw a less pronounced statistically significant decrease in total DOT. However, there was a statistically significant decrease in mean IV DOT and increase in mean PO DOT.

Although other ASP interventions made by a pharmacist were not captured in this study, the decrease in mean total DOT in region one may be attributed to these other ASP interventions found to be needed after reviewing the patient from the system list. Other ASP interventions may have included recommending discontinuation of antimicrobial therapy or recommendation of antimicrobials not targeted in the IV-to-PO initiative.

The findings of this study demonstrated overall cost savings in both regions. The cost savings were more pronounced in region one with the support of tele-ASP pharmacists’ reinforcement compared with region two. As ASPs are quality programs, overall cost benefit to the hospital is difficult to demonstrate.^17^ Cost level data was obtained for eight of the targeted antimicrobials, with linezolid being excluded from cost analysis as described above, and savings calculated based on the change in PO ratios. As these figures were estimated from the purchasing data, rather than charge data to the patients, our total amount is likely underestimated given the inability to quantify the monetary value of nursing time saved, avoidance of extra intravascular lines, and reduced LOS.^17^ Previous studies regarding the conversion of IV to PO antimicrobials in eligible patients demonstrate a sizable impact on total healthcare costs.^15,18^ Our study indicates a cost benefit from six months of data with the use of eight targeted antimicrobials. There is room for expansion to increase the number of targeted antimicrobials over a longer period of time, possibly resulting in a more drastic cost benefit in the long term.

A strength of this study includes the assessment of the same time frame from the year prior to the intervention to account for seasonal variances in antimicrobial usage. Additionally, this IV-to-PO protocol could be built into an EMR at other facilities for implementation as described in the methods. Another strength is the cost impact of implementing an IV-to-PO protocol. Alternatively, a limitation of the study is that EMR ASP IV-to-PO lists were not typically reviewed after regular work hours, and one of the sites in region one did not review the list on weekends. This may have resulted in delays of transition to PO or failure to capture patients eligible for conversion during that time. Additionally, multiple pharmacists at each regional hospital were responsible for reviewing the IV-to-PO lists. This may have introduced variation in the application of the inclusion and exclusion criteria of the protocol. Another limitation of the study would include that an extensive manual chart review was not conducted to collect LOS data or to determine which agents are most commonly intervened upon. Characteristics of the patients during each period, and other ASP interventions besides IV-to-PO interventions were not able to be collected in this study. Additionally, pricing may vary among institutions, introducing variability in cost savings. Importantly, it should be noted that we cannot definitively conclude that the ID pharmacist solely contributed to the success of the IV-to-PO program, as other confounding variables could have affected the overall benefit that were not evaluated. However, this cost saving initiative was launched by the ID pharmacist and the findings of this study aided in the addition of two FTEs to the ASP teams, including one FTE for region 2.

This study adds to the emerging body of literature for tele-ASP pharmacists’ impact. Our results emphasize the importance of an ID-trained pharmacist with dedicated FTEs in enhancing tele-ASPs and as an added benefit can provide significant cost savings as demonstrated in region one. Although region two did not have an ID-trained pharmacist’s oversight, region two also saw an improvement in each area evaluated. Certain community hospitals may not have resources dedicated to a full-time ID-trained pharmacist. Many facilities do not have a pharmacist with dedicated time to stewardship, or the pharmacist has numerous other clinical responsibilities. In these hospitals, optimizing EMR lists is still highly useful. A potential area of research could look at the specific impact in a patient’s hospital LOS.

## Conclusions

Our findings demonstrate the impact of tele-ASP pharmacists’ oversight in the implementation of an IV-to-PO protocol. The results indicate that this ASP intervention reduced IV DOTs, leading to improved IV-to-PO conversion rates, with region one exhibiting a more profound increase. This emphasizes the importance of ID-trained pharmacists with dedicated FTEs in enhancing ASP.
